# Trends of preterm birth and low birth weight in Japan: a one hospital-based study

**DOI:** 10.1186/1471-2393-12-162

**Published:** 2012-12-26

**Authors:** Takashi Yorifuji, Hiroo Naruse, Saori Kashima, Takeshi Murakoshi, Tsuguhiko Kato, Sachiko Inoue, Hiroyuki Doi, Ichiro Kawachi

**Affiliations:** 1 Department of Human Ecology, Okayama University Graduate School of Environmental and Life Science, 3-1-1 Tsushima-naka, Kita-ku, Okayama, 700-8530, Japan; 2 Department of Obstetrics, Seirei Hamamatsu General Hospital, 2-12-12 Sumiyoshi, Naka-ku, Hamamatsu, Shizuoka, Japan; 3 Department of Public Health and Health Policy, Hiroshima University Institute of Biomedical & Health Sciences, 1-2-3 Kasumi, Minami-ku, Hiroshima, Japan; 4 Department of Epidemiology, Okayama University Graduate School of Medicine, Dentistry and Pharmaceutical Sciences, 2-5-1 Shikata-cho, Kita-ku, Okayama, Japan; 5 Department of Society, Human Development, and Health, Harvard School of Public Health, 677 Huntington Avenue, Boston, MA, USA

**Keywords:** Caesarean section, Low birth weight, Pregnancy, Preterm birth, Reproductive health

## Abstract

**Background:**

The proportions of preterm birth (PTB, ie., delivered before 37 gestational weeks) and low birth weight (LBW, ie., birth weight less than 2500 g at delivery) have been rising in developed countries. We sought to examine the factors contributing to the rise in Japan, with particular focus on the effects of obstetric interventions.

**Methods:**

We used a database maintained by one large regional hospital in Shizuoka, Japan. We restricted the analysis to mothers who delivered live singleton births from 1997 to 2010 (n = 19,221). We assessed the temporal trends in PTB and LBW, then divided the study period into four intervals and compared the proportions of PTB and LBW. We also compared the newborns’ outcomes between the intervals.

**Results:**

PTB, in particular medically indicated PTB, increased considerably. The increase was largely explained by changes in caesarean sections. The neonatal outcomes did not worsen, and instead the Apgar scores and proportions requiring neonatal intensive care unit (NICU) admission improved. In particular, the risks of NICU admission in the interval from 2007 to 2010 were decreased among all births [odds ratio (OR): 0.84; 95% confidence interval (CI): 0.75, 0.95] and medically indicated births (OR: 0.44; 95% CI: 0.29, 0.68) compared with the interval from 1997 to 2000.

**Conclusions:**

Despite the increases in PTB as well as LBW, the present study suggests benefits of obstetric interventions. Rather than simple categorization of PTB or LBW, indicators such as perinatal mortality or other outcomes may be more appropriate for evaluation of perinatal health in developed countries.

## Background

Preterm birth (PTB, ie. delivered before 37 gestational weeks) and low birth weight (LBW, ie. birth weight less than 2500 g at delivery) are often used as markers for prematurity of newborns, and are associated with perinatal mortality as well as adverse consequences in later adulthood [1-3]. Despite increased knowledge of the risk factors, the proportions of PTB and LBW are increasing in developed countries (e.g., from 10.6% and 5.9% in 1990 to 12.2% and 6.4% in 2009 in the United States, respectively) [4]. Previous studies examined the factors contributing to these rises (in particular PTB), and found that PTB at gestational age from 34 to 36 weeks (late preterm) has been increasing [5,6]. Although the rise of PTB may be partly explained by changes in sociodemographic and behavioral factors, most of the studies indicated a role for obstetric interventions in this rise [7-11].

Paradoxically, despite the increases in PTB and LBW, the infant mortality and neonatal mortality rates have been declining in these developed countries [12]. These contrasting trends have raised questions about the simultaneous roles of obstetric interventions as both a contributor to the rise in PTB and to the improvements in PTB-associated mortality [5,7,13,14]. However, the number of studies addressing the issue of the benefits and risks of obstetric interventions is still limited. Moreover, most of the previous studies utilized birth certificates. Consequently, they could only evaluate the impacts on mortality and could not provide more detailed information in terms of biological indicators, such as the Apgar score and cord blood pHs.

Similarly in Japan, the proportions of PTB and LBW have been increasing (4.1% and 5.2% in 1980 to 5.7% and 9.6% in 2010, respectively), while the infant mortality and neonatal mortality rates have been declining steeply [15]. Surprisingly, Japan seems to be entering a new era characterized by lower neonatal mortality than post-neonatal mortality, in contrast to most developed countries [16]. In this highly advanced setting, the present study sought to examine the factors contributing to the rise in PTB as well as LBW. If obstetric interventions played important roles, consistent with previous studies, we sought to examine whether such interventions bring benefit or harm to newborns, especially in terms of biological indicators at birth.

## Methods

### Participants

Data were extracted from a perinatal database maintained since January 1997 at the Seirei Hamamatsu General Hospital, Shizuoka, Japan. The hospital is the largest tertiary hospital and main perinatal medical center in the western part of Shizuoka prefecture, and manages cases from low-risk to high-risk deliveries. The hospital reported 1,582 new live births in 2010 and about one-eighth of the babies born in the western part of Shizuoka were born in this hospital [17]. The database includes information on all of the mothers admitted to the Department of Obstetrics in the hospital (n = 21,855 from 1997 to 2010). In this study, we restricted the analysis to mothers who delivered live singleton births from January 1997 to December 2010, and defined the eligibility criteria for inclusion in the study as follows: singleton births; deliveries after 22 weeks of gestational age; and babies with Apgar scores of greater than one at one minute after birth. Using these criteria, we retrieved 19,221 births from the database.

### Definitions of PTB and LBW

We divided PTB by gestational age [18]: 22 to 27 weeks (extreme prematurity); 28 to 33 weeks (severe/moderate prematurity); and 34 to 36 weeks (late preterm). We also divided PTB at less than 37 weeks into medically indicated PTB and spontaneous PTB. Following a previous study [7], we defined medically indicated PTB as deliveries after caesarean section or medical induction prior to 37 weeks of gestation, and defined PTB other than medically indicated PTB as spontaneous PTB. The gestational ages were measured based on the last menstrual period, and were mostly confirmed or corrected by ultrasound measurements at about 10 weeks of gestational age [17].

We defined LBW as birth weight of less than 2500 g and term-LBW as LBW at term (born at more than 37 weeks of gestation).

### Obstetric variables and newborns’ outcomes

We retrieved information about obstetric variables and newborns’ outcomes from the perinatal database. The information included in the perinatal database was obtained once from the mothers by trained obstetricians or midwives at the time of the prenatal checkup when the expected due date was confirmed (about 10 weeks of gestational age), and added to or corrected at admission or delivery.

From the database, we collected information about whether the mothers had received fertility treatment, caesarean section, or medical induction (for the corresponding pregnancy). Moreover, we obtained the following markers for the newborns: Apgar score at one minute; Apgar score at five minutes; blood pH in the umbilical artery; blood pH in the umbilical vein; and neonatal intensive care unit (NICU) admission. The Apgar scores and blood pH were recorded using the same system throughout the study period [19]. With regard to blood gas measurements, blood was collected immediately after delivery by isolating a 10- to 20-cm segment of the cord, and the blood gas was measured immediately by an automatic blood gas analysis apparatus.

### Other covariate data

We also retrieved the following information from the database: maternal age at pregnancy; maternal height at pregnancy; maternal weight at pregnancy; maternal weight gain during pregnancy; maternal occupation (housewife, part-time worker, self-employed worker, employee, or professional worker); maternal alcohol intake during pregnancy (drinker or non-drinker); maternal smoking (never smoked, ex-smoker including mothers who quit smoking during pregnancy, or smoker); parity (0, 1, or ≥2); and paternal smoking (smoker or non-smoker). We calculated the body mass index (BMI) dividing the mother’s body weight before pregnancy (kg) by her height squared (m^2^).

### Statistical analyses

First, we assessed the temporal trends in the proportions of PTB and LBW (PTB less than 37 weeks, medically indicated PTB, spontaneous PTB, LBW, and term-LBW) from 1997 to 2010. We then divided the study period into four intervals (1997–2000, 2001–2003, 2004–2006, and 2007–2010). We compared the demographic characteristics as well as the proportions of PTB and LBW between the intervals. Next, we examined the covariates contributing to the discrepancy in the proportions of PTB (less than 37 weeks) and LBW in each interval.

Second, we compared the newborns’ outcomes between the study intervals among all births, medically indicated PTB, spontaneous PTB, PTB separated by gestational week, and LBW. We then estimated odds ratios (ORs) for the associations between the intervals and the NICU admissions, using the interval of 1997–2000 as a reference, by logistic regression. In the model, we adjusted for categorized maternal age (≤24.9, 25–34.9, or ≥35 years), parity, categorized maternal BMI (≤18.5, 18.6-24.9, or ≥25), maternal smoking, maternal occupation, maternal alcohol intake, and paternal smoking. These potential confounding factors were chosen a priori.

Finally, in sensitivity analyses, we separated births to mothers who experienced spontaneous onset of labor from the medically indicated PTBs and repeated the same analyses. Moreover, we used different intervals (1997–1999, 2000–2002, 2003–2004, 2005–2007, and 2008–2010) and repeated the same analyses as a robustness check.

All confidence intervals (CIs) were estimated at the 95% level. PASW software (version 18.0J; SPSS Japan Inc.) was used for the analyses.

Approval for this study was obtained from the Institutional Review Boards of Seirei Hamamatsu General Hospital and Okayama University.

## Results

**Figure 1:**
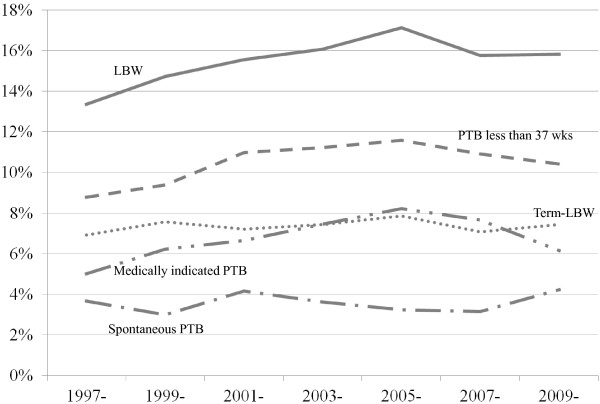
Trends of preterm birth (PTB) at less than 37 weeks, medically indicated PTB, spontaneous PTB, low birth weight (LBW), and term-LBW from 1997 to 2010.

Figure 1 shows the trends of each manifestation from 1997 to 2010. The proportions of PTB at less than 37 weeks, medically indicated PTB, and LBW increased with peaks around 2005 to 2006, while the proportions of term-LBW and spontaneous PTB showed little change during the study period.

The baseline characteristics of the newborns, mothers, and fathers (n = 19,221) separated by the intervals are shown in Table 1. As observed in Figure 1, PTB and LBW, in particular medically indicated PTB, increased and the peaks of the proportions were around the interval from 2004 to 2006. Among the increase of PTB at less than 37 weeks, although the absolute number of the increase was large for PTB at 34 to 36 weeks, PTB at each gestational week increased to some extent.

**Table 1:** Descriptive characteristics of newborns and parents

	**1997-2000** **(n = 5,515)**	**2001-2003** **(n = 3,927)**	**2004-2006** **(n = 4,025)**	**2007-2010** **(n = 5,754)**	**p value**^**§**^
**Newborn outcomes, n (%)**					
PTB less than 37 wk	501 (9.1)	412 (10.5)	484 (12.0)	614 (10.7)	<0.01
PTB 34 to 36 wk	309 (5.6)	256 (6.5)	272 (6.8)	367 (6.4)	0.10
PTB 28 to 33 wk	148 (2.7)	124 (3.2)	140 (3.5)	181 (3.1)	0.16
PTB 22 to 27 wk	44 (0.8)	32 (0.8)	72 (1.8)	66 (1.1)	<0.01
Medically indicated, less than 37 wk*	309 (5.6)	256 (6.5)	335 (8.3)	398 (6.9)	<0.01
Spontaneous, less than 37 wk*	184 (3.3)	150 (3.8)	141 (3.5)	212 (3.7)	0.60
LBW	774 (14.0)	601 (15.3)	691 (17.2)	908 (15.8)	<0.01
Term-LBW	399 (7.2)	292 (7.4)	305 (7.6)	417 (7.2)	0.91
Female sex, n (%)	2607 (47.3)	1903 (48.5)	1909 (47.4)	2831 (49.2)	0.22
**Parental variables**					
Maternal age (yr), mean (SD)	29.6 (4.4)	30 (4.7)	30.5 (4.7)	31.2 (4.9)	<0.01
≤24.9, n (%)	748 (13.6)	514 (13.1)	486 (12.1)	616 (10.7)	<0.01
25-34.9, n (%)	4128 (74.9)	2870 (73.1)	2863 (71.1)	3815 (66.3)	
≥35, n (%)	637 (11.6)	541 (13.8)	676 (16.8)	1323 (23.0)	
Maternal BMI^†^ at pregnancy, mean (SD)	20.6 (2.8)	20.6 (3.0)	20.8 (3.2)	20.9 (3.3)	<0.01
≤18.5, n (%)	1112 (20.2)	809 (20.7)	836 (20.9)	1165 (20.3)	<0.01
18.6-24.9, n (%)	4021 (73.1)	2820 (72.0)	2829 (70.7)	4056 (70.6)	
≥25, n (%)	371 (6.7)	287 (7.3)	334 (8.4)	524 (9.1)	
Maternal weight gain during pregnancy (kg), mean (SD)	8.8 (4.0)	8.8 (4.1)	9.2 (4.3)	9.6 (4.1)	<0.01
≤4.9 (%)	700 (14.2)	538 (15.4)	543 (14.4)	573 (10.3)	<0.01
5-9.9 (%)	2352 (47.6)	1598 (45.8)	1574 (41.7)	2361 (42.5)	
10-14.9 (%)	1632 (33.0)	1140 (32.7)	1380 (36.6)	2141 (38.5)	
≥15 (%)	260 (5.3)	212 (6.1)	278 (7.4)	481 (8.7)	
Maternal occupation, n (%)					
Professional worker	408 (7.5)	337 (8.7)	325 (8.4)	505 (10.1)	<0.01
Employee	1175 (21.6)	905 (23.4)	1005 (26.0)	1548 (31.1)	
Self-employed worker	65 (1.2)	46 (1.2)	39 (1.0)	45 (0.9)	
Part-time worker	155 (2.9)	177 (4.6)	136 (3.5)	213 (4.3)	
Housewife	3627 (66.8)	2407 (62.2)	2362 (61.1)	2670 (53.6)	
Maternal alcohol intake (drinkers), n (%)	384 (7.1)	215 (5.6)	143 (3.6)	129 (2.3)	<0.01
Maternal smoking, n (%)					
Never smoked	5210 (95.8)	3648 (94.4)	3747 (94.5)	5428 (94.7)	<0.01
Ex-smoker^‡^	54 (1.0)	75 (1.9)	98 (2.5)	154 (2.7)	
Smoker (during pregnancy)	176 (3.2)	142 (3.7)	122 (3.1)	147 (2.6)	
Fertility treatment	597 (10.8)	391 (10)	520 (12.9)	884 (15.4)	<0.01
Caesarean section	1253 (22.7)	999 (25.4)	1170 (29.1)	1648 (28.6)	<0.01
Induction	1637 (30.9)	1020 (27.4)	927 (23.7)	1386 (24.7)	<0.01
Parity (%)					
One	3023 (54.8)	2233 (56.9)	2280 (56.6)	3165 (55.1)	0.02
Two	1911 (34.7)	1282 (32.6)	1372 (34.1)	1924 (33.5)	
Three or more	581 (10.5)	412 (10.5)	373 (9.3)	651 (11.3)	
Paternal smoking, n (%)	2696 (51.4)	1784 (48.2)	1656 (42.8)	1960 (35.6)	<0.01

The newborns were live singleton births from January 1997 to December 2010 in a perinatal hospital in Hamamatsu, Japan (n = 19,221).

*Sum of medically indicated PTB and spontaneous PTB may not equal PTB less than 37 weeks because of missing data for delivery or induction variables.

^†^Body mass index was calculated as body weight (kg) divided by height squared (m^2^).

^‡^Ex-smoker included mothers who quit smoking during pregnancy.

^§^Obtained by the chi-square test or analysis of variance.

*BMI* body mass index; *LBW* low birth weight; *PTB* preterm birth, *SD* standard deviation.

During the study period, the mean maternal age increased and mothers who were older than 35 years increased from 11.6% in the period of 1997 to 2000 to 23.0% in the interval from 2007 to 2010 (Table 1). In addition, the rates of mothers with BMI of more than 25 tended to increase, and the proportion of mothers with adequate weight gain during pregnancy also increased during the period. While the number of parental smokers decreased, the number of maternal ex-smokers increased. With regard to obstetric interventions, the proportions of mothers experiencing fertility treatment and caesarean section increased during the period.

Compared with the years 1997–2000, PTB at less than 37 weeks increased by 1.37 times in the years 2004–2006 and 1.20 times in the years 2007–2010 (Additional file 1: Table S1). Maternal characteristics such as age, BMI at pregnancy, smoking, occupation, and alcohol intake explained the discrepancy, but medical interventions, in particular caesarean section, contributed the most to the increase in PTB at less than 37 weeks. We found similar tendencies for LBW. Some maternal characteristics such as smoking and alcohol intake attenuated the discrepancy, but the attenuations were largest for caesarean section.

Table 2 shows the Apgar scores, cord blood pHs (artery and vein), and percentages of NICU admission during the study period in all births and separated by each manifestation. None of the neonatal outcomes showed a trend in a worse direction, even among medically indicated PTBs, and instead the Apgar scores and rates of NICU admission improved over time.

**Table 2:** Apgar scores, cord blood pHs (artery and vein), and percentages of NICU admission

	**1997-2000**	**2001-2003**	**2004-2006**	**2007-2010**	p value*
**All births**					
Apgar score 1, mean (SD)	7.8 (1.4)	7.9 (1.3)	8 (1.2)	8 (1.2)	<0.01
Apgar score 5, mean (SD)	8.6 (0.9)	8.7 (0.9)	8.8 (0.8)	8.8 (0.7)	<0.01
Umbilical artery pH, mean (SD)	7.30 (0.07)	7.31 (0.07)	7.30 (0.07)	7.30 (0.07)	<0.01
Umbilical vein pH, mean (SD)	7.33 (0.07)	7.34 (0.08)	7.35 (0.07)	7.34 (0.07)	<0.01
NICU admission (%)	809 (15.2)	602 (15.8)	522 (13)	745 (12.9)	<0.01
**PTB**					
**Separated by type**					
**Medically indicated PTB, 37 wk**					
Apgar score 1, mean (SD)	5.3 (2.8)	5.8 (2.7)	6.5 (2.4)	6.5 (2.4)	<0.01
Apgar score 5, mean (SD)	7.6 (1.8)	7.5 (2.1)	8.3 (1.7)	8.2 (1.6)	<0.01
Umbilical artery pH, mean (SD)	7.29 (0.11)	7.30 (0.09)	7.30 (0.10)	7.29 (0.09)	0.40
Umbilical vein pH, mean (SD)	7.32 (0.10)	7.32 (0.09)	7.33 (0.1)	7.32 (0.10)	0.41
NICU admission (%)	256 (84.5)	214 (85.3)	244 (72.8)	287 (72.1)	<0.01
**Spontaneous PTB, 37 wk**					
Apgar score 1, mean (SD)	7.1 (1.9)	7.3 (2)	7.3 (1.8)	7.3 (2.1)	0.74
Apgar score 5, mean (SD)	8.1 (1.5)	8.4 (1.2)	8.6 (1.1)	8.5 (1.4)	<0.01
Umbilical artery pH, mean (SD)	7.31 (0.08)	7.33 (0.08)	7.3 (0.10)	7.32 (0.08)	0.03
Umbilical vein pH, mean (SD)	7.34 (0.09)	7.35 (0.09)	7.35 (0.13)	7.35 (0.10)	0.89
NICU admission (%)	112 (62.6)	89 (59.7)	77 (54.6)	141 (66.5)	0.15
**Separated by gestational week**					
**PTB 34–36 wk**					
Apgar score 1, mean (SD)	7.1 (1.9)	7.3 (1.9)	7.6 (1.6)	7.5 (1.7)	<0.01
Apgar score 5, mean (SD)	8.3 (1.2)	8.5 (1.1)	8.7 (1.1)	8.7 (1)	<0.01
Umbilical artery pH, mean (SD)	7.3 (0.1)	7.31 (0.08)	7.3 (0.08)	7.3 (0.07)	0.28
Umbilical vein pH, mean (SD)	7.33 (0.09)	7.34 (0.09)	7.34 (0.1)	7.34 (0.08)	0.58
NICU admission (%)	191 (63.2)	161 (63.6)	146 (53.7)	212 (57.8)	0.05
**PTB 28–33 wk**					
Apgar score 1, mean (SD)	4.6 (2.8)	5.4 (2.6)	6.6 (2)	6.3 (2.5)	<0.01
Apgar score 5, mean (SD)	7.4 (1.6)	7.4 (1.9)	8.5 (1.4)	8.2 (1.5)	<0.01
Umbilical artery pH, mean (SD)	7.31 (0.09)	7.32 (0.09)	7.31 (0.1)	7.3 (0.11)	0.52
Umbilical vein pH, mean (SD)	7.33 (0.1)	7.33 (0.09)	7.34 (0.1)	7.33 (0.12)	0.95
NICU admission (%)	142 (97.3)	117 (95.9)	112 (80)	157 (86.7)	<0.01
**PTB 22–27 wk**					
Apgar score 1, mean (SD)	2.7 (1.9)	2.6 (2.1)	3.8 (2.3)	3.7 (2.5)	<0.01
Apgar score 5, mean (SD)	5.1 (2.4)	4.6 (2.5)	7.2 (2.6)	6.8 (2.4)	<0.01
Umbilical artery pH, mean (SD)	7.28 (0.12)	7.32 (0.07)	7.28 (0.14)	7.28 (0.12)	0.59
Umbilical vein pH, mean (SD)	7.28 (0.11)	7.31 (0.08)	7.31 (0.15)	7.29 (0.14)	0.78
NICU admission (%)	38 (90.5)	28 (90.3)	68 (94.4)	63 (95.5)	0.65
**LBW**					
Apgar score 1, mean (SD)	6.7 (2.4)	6.9 (2.3)	7.1 (2.1)	7.2 (2.1)	<0.01
Apgar score 5, mean (SD)	8.2 (1.5)	8.2 (1.6)	8.6 (1.4)	8.5 (1.3)	<0.01
Umbilical artery pH, mean (SD)	7.30 (0.09)	7.31 (0.08)	7.30 (0.09)	7.30 (0.08)	<0.01
Umbilical vein pH, mean (SD)	7.33 (0.09)	7.33 (0.09)	7.34 (0.10)	7.33 (0.09)	0.40
NICU admission (%)	447 (59.4)	380 (64.3)	385 (55.7)	525 (57.8)	0.02

*LBW* low birth weight; *NICU* neonatal intensive care unit; *PTB* preterm birth; *SD* standard deviation.

*Obtained by the chi-square test or analysis of variance.

Even after adjustment for the covariates (Table 3), the risks of NICU admission in the interval from 2007 to 2010 were decreased among all births (OR: 0.84; 95%CI: 0.75, 0.95), medically indicated births (OR: 0.44; 95%CI: 0.29, 0.68), and PTB at 28 to 33 weeks (OR: 0.14; 95%CI: 0.04, 0.50) compared with the interval from 1997 to 2000.

**Table 3:** Adjusted odds ratios and corresponding 95% confidence intervals* for NICU admissions

	**1997-2000**	**2001-2003**	**2004-2006**	**2007-2010**
**All births**	1 (Ref)	1.06 (0.94, 1.19)	0.81 (0.71, 0.92)	0.84 (0.75, 0.95)
**PTB**				
**Separated by type**				
Medically indicated PTB 37 wk	1 (Ref)	1.12 (0.67, 1.87)	0.47 (0.30, 0.72)	0.44 (0.29, 0.68)
Spontaneous PTB 37 wk	1 (Ref)	0.80 (0.49, 1.29)	0.64 (0.39, 1.05)	1.05 (0.66, 1.65)
**Separated by gestational week**				
PTB 34–36 wk	1 (Ref)	1.02 (0.70, 1.47)	0.67 (0.47, 0.96)	0.77 (0.54, 1.09)
PTB 28–33 wk	1 (Ref)	0.57 (0.12, 2.67)	0.07 (0.02, 0.26)	0.14 (0.04, 0.50)
PTB 22–27 wk	1 (Ref)	0.77 (0.09, 6.56)	1.57 (0.21, 11.48)	5.22 (0.38, 71.55)
**LBW**	1 (Ref)	1.24 (0.98, 1.56)	0.84 (0.67, 1.05)	0.97 (0.78, 1.20)

*Adjusted for maternal age, parity, maternal body mass index, maternal smoking, maternal occupation, maternal alcohol, and paternal smoking.

*LBW*, low birth weight; *PTB*, preterm birth.

In sensitivity analyses, we separated the 194 (15.4%) mothers who experienced spontaneous onset of labor from the medically indicated PTBs. Our conclusions were not affected by the exclusion, e.g., the OR for NICU admission in the interval from 2007 to 2010 was 0.48 (95%CI: 0.30, 0.78) among the medically indicated PTB (excluding spontaneous onset of labor). Furthermore, our conclusions were not affected when we used alternative intervals (1997–1999; 2000–2002; 2003–2004; 2005–2007; and 2008–2010).

## Discussion

In the present study, we explored the trends in PTB and LBW in a perinatal center in Japan, and examined the contributing factors and changes in neonatal outcomes. Although term-LBW did not increase, PTB, in particular medically indicated PTB, increased considerably. These increases were largely explained by changes in obstetric interventions. Despite the increases, the neonatal outcomes did not worsen, and instead the Apgar scores and proportions of NICU admission improved.

The proportions of PTB and LBW increased with peaks around 2005 in the present study, consistent with the national vital statistics in Japan (4.1% and 5.2% in 1980 to 5.7% and 9.6% in 2010, respectively) [15]. In contrast, term-LBW did not increase, indicating that the observed increase in LBW can probably be explained by the increase in earlier deliveries (i.e., PTB). The finding that the increase in medically indicated PTB was larger than that in spontaneous PTB was consistent with previous studies in western countries [7,11,14,20]. Although the increase in PTB in western countries has been almost entirely among the late preterm (34 to 36 weeks) [5,6,21], the increase in PTB at less than 33 weeks also accounted for a small fraction of the increase in total PTB in the present study, consistent with the national vital statistics in Japan. For example, about 14% of the increase in total PTB was explained by the increase in PTB at equal to or less than 31 weeks in Japan [15].

The increase in PTB was largely explained by changes in obstetric interventions, in particular caesarean sections, followed by changes in maternal sociodemographic and behavioral factors. Indeed, the proportion of caesarean sections has been steadily rising in Japan (14.7% for hospitals and 9.9% for clinics in 1996 to 23.3% and 13.0% in 2008, respectively) [22]. Although the changes in sociodemographic and behavioral factors may directly contribute to the rise in the proportion of caesarean sections, obstetricians may also be prone to conduct caesarean sections in a more proactive manner, independent of such demographic changes. In Japan, people consider that the lean structure of women and reduced weight gain during pregnancy might contribute to the rise in LBW [23], because low BMI and poor weight gain are risk factors for LBW [24]. However, in the present study, obese mothers increased and mothers recently showed more adequate weight gain, and thus the increases in LBW as well as PTB could not be explained by these factors.

Despite the increases in PTB and LBW, the neonatal outcomes did not worsen, and instead showed improvement. Previous studies also indicated the benefits of obstetric interventions on perinatal and neonatal mortality [5,7]. The present study supported these previous studies by providing further findings for the benefits on biological indicators (Apgar score and cord blood pHs). Accordingly, it can be inferred that obstetricians may intervene appropriately (i.e., conduct necessary interventions at appropriate periods), which may contribute to low infant mortality and neonatal mortality rates (2.29 and 1.09 per 1000 births in 2010, respectively) in Japan [15], in combination with advancement of NICU facilities. However, it should be noted that, since the prognostic outcomes of late preterm births are reported to be worse than those of term newborns in general [25], further research should be conducted to evaluate whether such obstetric interventions lead to better prognostic outcomes in newborns [26].

One of the strengths of the present study is that we could utilize the biological indicators from a clinical dataset, compared with the previous studies using birth certificates. On the other hand, there is a problem with generalizability of the findings. As described in the Methods section, not all of the babies in the western part of Shizuoka are born in this particular hospital. In addition, the hospital is the main perinatal center in the area, and therefore manages not only low-risk but also high-risk deliveries. As a consequence, the proportions of PTB and LBW were higher than those reported nationally, as expected. Thus, it is possible that this hospital-based sampling method may affect the external validity. However, the present findings are consistent with previous studies based on general populations conducted in western countries, and thus the main findings would not be affected considerably.

The methods for measuring gestational weeks, obtaining Apgar scores, and evaluating blood gas were standardized throughout the study period. Moreover, the standard of NICU admission in this institution did not change. Thus, changes in obstetric diagnoses or techniques during the study period may not affect the present findings.

We could not obtain individual socioeconomic status variables (education or income) other than occupation. Therefore, other factors that we did not consider in the analyses might explain the discrepancy to some extent. However, since we adjusted for various variables that may define parental socioeconomic status or other variables, the discrepancy generated from these factors would not be larger than that for caesarean sections.

## Conclusion

Despite the increases in PTB as well as LBW, the present study suggests the benefits of obstetric interventions (i.e., careful management of pregnant mothers and appropriate interventions). Rather than simple categorization of PTB or LBW, indicators such as perinatal mortality or biological indicators may be crucial for evaluation of perinatal health in developed countries. Future studies examining the prognostic outcomes of newborns delivered after obstetric interventions are warranted.

## Abbreviations

BMI: Body mass index; CI: Confidence interval; LBW: Low birth weight; NICU: Neonatal intensive care unit; OR: Odds ratio; PTB: Preterm birth.

## Competing interests

The authors declared that they have no conflict of interests.

## Authors’ contributions

Study concept and design: TY, SK, TK, SI, IK; Data collection: HN,TM, HD; Data handling: TY, SK, SI; Analysis: TY; Interpretation of data: TY, SK, TK, SI, IK; Drafting of the manuscript: TY, IK; Critical revision of the manuscript: SK, TK, SI, HD; Study supervision: IK. All authors read and approved the final manuscript.

## Pre-publication history

The pre-publication history for this paper can be accessed here:

http://www.biomedcentral.com/1471-2393/12/162/prepub

## Supplementary Material

Additional file 1: Table S1 Online Table. Crude and adjusted interval odds ratios* (and corresponding 95% CI) for PTB less than 37 wks and LBW. Click here for file

## References

[B1] Baker JL Olsen LW Sorensen TI Weight at birth and all-cause mortality in adulthood Epidemiology 2008 19 197 203 10.1097/EDE.0b013e31816339c6 18300695

[B2] Carlo WA Carlo WA, Kilegman RM Overview of Mortality and Morbidity Nelson Textbook of Pediatrics 2011 19

[B3] Saigal S Doyle LW An overview of mortality and sequelae of preterm birth from infancy to adulthood Lancet 2008 371 261 269 10.1016/S0140-6736(08)60136-1 18207020

[B4] U.S. Dept. of Health and Human Services Births: Final data for 2009 National Vital Statistics Reports 2011 60 1 72 22670489

[B5] Joseph KS Demissie K Kramer MS Obstetric intervention, stillbirth, and preterm birth Semin Perinatol 2002 26 250 259 10.1053/sper.2002.34769 12211615

[B6] MacDorman MF Declercq E Zhang J Obstetrical intervention and the singleton preterm birth rate in the United States from 1991–2006 Am J Public Health 2010 100 2241 2247 10.2105/AJPH.2009.180570 20864720 PMC2951941

[B7] Ananth CV Joseph KS Oyelese Y Demissie K Vintzileos AM Trends in preterm birth and perinatal mortality among singletons: United States, 1989 through 2000 Obstet Gynecol 2005 105 1084 1091 10.1097/01.AOG.0000158124.96300.c7 15863548

[B8] Barros FC Velez Mdel P Temporal trends of preterm birth subtypes and neonatal outcomes Obstet Gynecol 2006 107 1035 1041 10.1097/01.AOG.0000215984.36989.5e 16648408

[B9] Joseph KS Kramer MS Marcoux S Ohlsson A Wen SW Allen A Platt R Determinants of preterm birth rates in Canada from 1981 through 1983 and from 1992 through 1994 New Engl J Med 1998 339 1434 1439 10.1056/NEJM199811123392004 9811918

[B10] Kramer MS Platt R Yang H Joseph KS Wen SW Morin L Usher RH Secular trends in preterm birth: a hospital-based cohort study JAMA 1998 280 1849 1854 10.1001/jama.280.21.1849 9846780

[B11] VanderWeele TJ Lantos JD Lauderdale DS Rising preterm birth rates, 1989–2004: changing demographics or changing obstetric practice? Soc Sci Med 2012 74 196 201 10.1016/j.socscimed.2011.10.031 22177849 PMC3259145

[B12] World Health Organization World Health Statistics 2011 2011 Geneva: World Health Organization

[B13] Basso O Rasmussen S Weinberg CR Wilcox AJ Irgens LM Skjaerven R Trends in fetal and infant survival following preeclampsia JAMA 2006 296 1357 1362 10.1001/jama.296.11.1357 16985227

[B14] Lisonkova S Hutcheon JA Joseph KS Temporal trends in neonatal outcomes following iatrogenic preterm delivery BMC Pregnancy Childbirth 2011 11 39 10.1186/1471-2393-11-39 21612655 PMC3130708

[B15] Vital Statisticshttp://www.mhlw.go.jp/toukei/list/81-1.html

[B16] Yorifuji T Tanihara S Inoue S Takao S Kawachi I The role of medicine in the decline of post-War infant mortality in Japan Paediatr Perinat Ep 2011 25 601 608 10.1111/j.1365-3016.2011.01216.x 21980949

[B17] Yorifuji T Naruse H Kashima S Ohki S Murakoshi T Takao S Tsuda T Doi H Residential proximity to major roads and preterm births Epidemiology 2011 22 74 80 10.1097/EDE.0b013e3181fe759f 21052006

[B18] Goldenberg RL Culhane JF Iams JD Romero R Epidemiology and causes of preterm birth Lancet 2008 371 75 84 10.1016/S0140-6736(08)60074-4 18177778 PMC7134569

[B19] Cunningham FG Williams JW Williams obstetrics 2010 23 New York: McGraw-Hill Medical

[B20] Norman JE Morris C Chalmers J The effect of changing patterns of obstetric care in Scotland (1980–2004) on rates of preterm birth and its neonatal consequences: perinatal database study PLoS Med 2009 6 e1000153 10.1371/journal.pmed.1000153 19771156 PMC2740823

[B21] Davidoff MJ Dias T Damus K Russell R Bettegowda VR Dolan S Schwarz RH Green NS Petrini J Changes in the gestational age distribution among U.S. singleton births: impact on rates of late preterm birth, 1992 to 2002 Semin Perinatol 2006 30 8 15 10.1053/j.semperi.2006.01.009 16549207

[B22] Basic Statistics on Regional Public Health and Medical Carehttp://www.mhlw.go.jp/toukei/list/142-1.html

[B23] Nakamura T Current state and background of birth weight reduction] (in Japanese) Shonika Rinsho 2011 64 2274 2285

[B24] Siega-Riz AM Viswanathan M Moos MK Deierlein A Mumford S Knaack J Thieda P Lux LJ Lohr KN A systematic review of outcomes of maternal weight gain according to the Institute of Medicine recommendations: birthweight, fetal growth, and postpartum weight retention Am J Obstet Gynecol 2009 201 339 e331-314 19788965 10.1016/j.ajog.2009.07.002

[B25] Kramer MS Demissie K Yang H Platt RW Sauve R Liston R The contribution of mild and moderate preterm birth to infant mortality. Fetal and Infant Health Study Group of the Canadian Perinatal Surveillance System JAMA 2000 284 843 849 10.1001/jama.284.7.843 10938173

[B26] Wilcox AJ Fertility and pregnancy: an epidemiologic perspective 2010 Oxford; New York: Oxford University Press

