# Supplementary material for: Trends of preterm birth and low birth weight in Japan: a one hospital-based study
Source: BMC Pregnancy Childbirth. 2012 Dec 26;12:162. doi: 10.1186/1471-2393-12-162 (PMC3562268; doi:10.1186/1471-2393-12-162)
Supplement: Additional file 1: Table S1 — Online Table. Crude and adjusted interval odds ratios* (and corresponding 95% CI) for PTB less than 37 wks and LBW. [file 1471-2393-12-162-S1.docx]

| Online Table. Crude and adjusted interval odds ratios* (and corresponding 95% CI) for PTB less than 37 wks and LBW. | | | | | |
| --- | --- | --- | --- | --- | --- |
|  |  | 1997-2000 | 2001-2003 | 2004-2006 | 2007-2010 |
| PTB less than 37 wks | |  |  |  |  |
|  | Crude | 1 (Ref) | 1.17 (1.02,1.35) | 1.37 (1.20,1.56) | 1.20 (1.06,1.35) |
|  |  |  |  |  |  |
|  | Adjusted for categorized age | 1 (Ref) | 1.17 (1.02,1.35) | 1.36 (1.19,1.55) | 1.18 (1.04,1.34) |
|  | Adjusted for parity | 1 (Ref) | 1.17 (1.02,1.35) | 1.37 (1.20,1.57) | 1.19 (1.06,1.35) |
|  | Adjusted for categorized BMI | 1 (Ref) | 1.17 (1.02,1.34) | 1.33 (1.17,1.52) | 1.19 (1.05,1.35) |
|  | Adjusted for maternal smoking | 1 (Ref) | 1.16 (1.01,1.33) | 1.34 (1.17,1.53) | 1.19 (1.05,1.35) |
|  | Adjusted for maternal occupation | 1 (Ref) | 1.17 (1.02,1.34) | 1.34 (1.17,1.53) | 1.23 (1.08,1.40) |
|  | Adjusted for categorized weight gain during pregnancy | 1 (Ref) | 1.26 (1.08,1.47) | 1.53 (1.32,1.77) | 1.52 (1.32,1.74) |
|  | Adjusted for alcohol | 1 (Ref) | 1.17 (1.02,1.34) | 1.35 (1.18,1.55) | 1.19 (1.05,1.35) |
|  | Adjusted for fertility treatment | 1 (Ref) | 1.16 (1.01,1.34) | 1.36 (1.19,1.55) | 1.19 (1.05,1.35) |
|  | Adjusted for caesarean section | 1 (Ref) | 1.13 (0.98,1.30) | 1.24 (1.09,1.43) | 1.09 (0.96,1.24) |
|  | Adjusted for induction | 1 (Ref) | 1.16 (1.01,1.34) | 1.33 (1.16,1.52) | 1.17 (1.03,1.33) |
|  | Adjusted for paternal smoking | 1 (Ref) | 1.21 (1.04,1.39) | 1.43 (1.25,1.64) | 1.25 (1.10,1.42) |
|  |  |  |  |  |  |
| LBW | |  |  |  |  |
|  | Crude | 1 (Ref) | 1.11 (0.99,1.24) | 1.27 (1.14,1.42) | 1.15 (1.03,1.27) |
|  |  |  |  |  |  |
|  | Adjusted for categorized age | 1 (Ref) | 1.11 (0.99,1.24) | 1.27 (1.13,1.42) | 1.14 (1.03,1.27) |
|  | Adjusted for parity | 1 (Ref) | 1.10 (0.98,1.24) | 1.27 (1.14,1.42) | 1.15 (1.03,1.27) |
|  | Adjusted for categorized BMI | 1 (Ref) | 1.10 (0.98,1.24) | 1.25 (1.12,1.40) | 1.15 (1.03,1.27) |
|  | Adjusted for maternal smoking | 1 (Ref) | 1.10 (0.98,1.23) | 1.24 (1.11,1.39) | 1.14 (1.03,1.27) |
|  | Adjusted for maternal occupation | 1 (Ref) | 1.11 (0.99,1.24) | 1.24 (1.11,1.39) | 1.16 (1.04,1.29) |
|  | Adjusted for categorized weight gain during pregnancy | 1 (Ref) | 1.21 (1.07,1.38) | 1.42 (1.25,1.6) | 1.40 (1.25,1.57) |
|  | Adjusted for alcohol | 1 (Ref) | 1.11 (0.98,1.24) | 1.25 (1.11,1.40) | 1.14 (1.02,1.26) |
|  | Adjusted for fertility treatment | 1 (Ref) | 1.10 (0.98,1.24) | 1.26 (1.13,1.41) | 1.15 (1.04,1.28) |
|  | Adjusted for caesarean section | 1 (Ref) | 1.07 (0.95,1.20) | 1.17 (1.04,1.31) | 1.06 (0.95,1.18) |
|  | Adjusted for induction | 1 (Ref) | 1.09 (0.96,1.22) | 1.23 (1.10,1.38) | 1.11 (1.00,1.24) |
|  | Adjusted for paternal smoking | 1 (Ref) | 1.15 (1.02,1.29) | 1.3 (1.16,1.46) | 1.19 (1.07,1.33) |
|  |  |  |  |  |  |
| * We used the interval of 1997-2000 as a reference and estimated odds ratios (ORs) for PTB and LBW. We entered each covariate separately into the models and examined how the adjustment moved the crude ORs. | | | | | |
| BMI: body mass index, CI: confidence interval, LBW: low birth weight, PTB: preterm birth | | | | | |
